# Peer Review of “Cross-Modal Sensory Boosting to Improve High-Frequency Hearing Loss: Device Development and Validation”

**DOI:** 10.2196/55727

**Published:** 2024-02-09

**Authors:** ­ Anonymous

**Keywords:** audiology, hearing, high-frequency, wristband, develop, development, wearable, wearables, machine learning, phoneme, phonemes, hear, vibrotactile, vibration, vibrations, sound, sounds, hearing loss, loud noise, loud noises, noise pollution, hearing aids, hearing aid

*This is the peer-review report for “Cross-Modal Sensory Boosting to Improve High-Frequency Hearing Loss: Device Development and Validation”.*

## Round 1 Review

### General Comments

This paper [1] highlights the utility and perceived communication benefits of the Clarity vibrotactile band for users with high-frequency hearing loss. Overall, this is a well-designed study that demonstrates the effectiveness of this assistive listening device that provides benefits for listeners with high-frequency hearing loss in complex listening situations as measured by the Abbreviated Profile of Hearing Aid Benefit (APHAB). Additionally, this study provides subjective evidence that both hearing aid (HA) users and non-HA users experience benefit from the Clarity device. Specifically, the non-HA users report more benefits across different listening conditions (background noise and reverberation) than HA users.

### Specific Comments

#### Major Comments

1. Consider referencing Glick and Sharma [2] in your Introduction as it relates to the cross-modal plasticity associated with age-related hearing loss (presbycusis).
2. In the Methods section, consider starting with a clear description of the participants. Who are they, how many, how many were HA users versus non-HA users, age, etc. While the majority of this information is embedded later in the article, it is not readily accessible.
3. In the Methods section, consider creating a subheading or table for the audiometric data of the participants and including additional information like a description of their audiometric data (type, degree, configuration), pure tone average (500, 1000, and 2000 Hz), symmetry of the hearing loss, how many were considered to be within normal limits up to 2000 Hz versus having hearing loss at lower frequencies (≤2000 Hz). This could have a significant impact on speech understanding difficulties, especially in complex listening environments.
4. For the audiometric data, how many participants provided their test results from a doctor of audiology or hearing health care professional? How many provided results from the mobile app? Is it possible to confirm that all participants had sensorineural hearing loss and not mixed or conductive hearing loss?
5. In the Device subsection, consider adding additional information regarding the microphone characteristics. Additionally, define “GRMS.”
6. In the Algorithm subsection, you mention the sham algorithm and the /f/ motor. In the sham condition, which motor represents the /f/ phoneme, and which additional phonemes are used in the sham condition?
7. Additionally, the sham condition is never mentioned in the Results or Discussion. Consider adding this information to the manuscript, or if you choose not to, consider not introducing the sham algorithm.
8. In Figure 3, consider changing the y-axis to “APHAB Score (%)” and refer to the APHAB benefit scores as scores or percentages instead of points in the text.
9. For the simple linear regression, consider adding a statement that indicates what this means or its importance.
10. In Figure 5, consider adding bars for weeks 0 and 1 to help readers visualize the results in the text.
11. Consider creating a line graph that highlights the greater decrease in APHAB scores from baseline to week 6 for those without HAs than those with HAs (as discussed in the Results).
12. In Figure 6, this figure represents benefit scores from baseline (wk 0) to week 6, correct? Consider clarifying the figure text and removing the information regarding the subgroups.
13. In the Discussion and Conclusion sections, I do not think it is accurate to say that the Clarity device “improved their understanding of speech communication” because that was not what was measured. The APHAB is a subjective measure, which to me means that all the benefits users received from using the Clarity are perceived benefits and are not measurable improvements in understanding. To claim speech understanding improvements, I feel you would need to document that through an objective speech understanding measure such as the word recognition score in quiet, word recognition score in noise, Quick Speech in Noise, etc.
14. In the Discussion section, you refer to the group with a higher APHAB score experiencing a greater improvement. Is this the group that uses HAs, or is this a different subgroup? It would be interesting to know how many in this group had hearing loss between 250-2000 Hz.
15. In the Discussion section, you report subgroup data for background noise, reverberation, and ease of communication that is not documented or reported in the Results section or any figures/tables. Consider adding this.
16. In the Conclusion section, you mention that “results also demonstrate that individuals who had the greatest amount of difficulty understanding speech prior to.” Is this the without HA subgroup or a different subgroup? A few times throughout the article, these labels appear to be used interchangeably. While this may be accurate for your data set, I would caution that these terms/labels are not mutually exclusive.

#### Minor Comments

1. In the Introduction, the authors mention that HA and cochlear implant users commonly report disappointment with understanding speech and reference Hickson et al [3]. While this could be true, the majority of users’ complaints are specifically related to difficulties understanding speech in complex or noisy listening environments, not just in quiet as is implied.
2. How much were participants compensated for their participation?
3. In Figure 2, I assume your scale for the y-axis is dB of HL? Consider clarifying which dB scale was used.
4. In the Paradigms subsection, does the Clarity device have any data logging features that can objectively record how often or how long the participant is using the device or in what listening conditions the user is in with the device (eg, quiet rooms, noisy restaurants, or reverberant auditoriums)?
5. In the APHAB subsection, consider rewording for clarity: “modified version of the Abbreviated Profile of Hearing Aid Benefit (APHAB) which did not include six questions related to the aversiveness subscale (Cox, 1997).”
6. In the Results section, consider rewording for clarity: “...they ended the study at a lower level of disability than those with hearing aids.”
7. The implication of microphone location briefly mentioned in the Discussion is very important in my opinion. Microphone location is a significant issue even for ear-level HAs. I can only imagine the microphone placement significantly impacts the benefit and utility of the Clarity.
8. In the Conclusion section, consider rewording for clarity: “We found that while both hearing aid and non-hearing aid users with high frequency hearing loss reported benefited, vibrotactile feedback appears to be more beneficial for non-hearing aid users.”
9. The manuscript does not include an ethical approval statement or a limitations section.

## Abbreviations

APHAB: Abbreviated Profile of Hearing Aid Benefit
HA: hearing aid

## References

[1] Kohler I, Perrotta MV, Ferreira T, Eagleman DM (2024). Cross-modal sensory boosting to improve high-frequency hearing loss: device development and validation. JMIRx Med.

[2] Glick H, Sharma A (2017). Cross-modal plasticity in developmental and age-related hearing loss: clinical implications. Hear Res.

[3] Hickson L, Meyer C, Lovelock K, Lampert M, Khan A (2014). Factors associated with success with hearing aids in older adults. Int J Audiol.

